# A Spatial Metabolomics Annotation Workflow Leveraging Cyclic Ion Mobility and Machine Learning-Predicted Collision Cross Sections

**DOI:** 10.1021/jasms.5c00090

**Published:** 2025-05-21

**Authors:** Dmitry Leontyev, Eric C. Gier, Viraj A. Master, Rebecca S. Arnold, John A. Petros, Facundo M. Fernández

**Affiliations:** † School of Chemistry and Biochemistry, 1372 Georgia Institute of Technology, Atlanta, Georgia 30332, United States; ‡ Winship Cancer Institute, Emory University, Atlanta, Georgia 30322, United States; § Department of Urology, 12239 Emory School of Medicine, Atlanta, Georgia 30342, United States; ∥ Parker H. Petit Institute for Bioengineering and Bioscience, Atlanta, Georgia 30332, United States

## Abstract

In nontargeted spatial metabolomics, accurate annotation is crucial for understanding metabolites’ biological roles and spatial patterns. MS^2^ mass spectrometry imaging (MSI) coverage is often incomplete or nonexistent, resulting in many unknown features that represent an untapped source of biological information. Ion mobility-derived collision cross sections (CCS) have been leveraged as valuable descriptors for confirming putative metabolite annotations, distinguishing isomers, and aiding in unknown structural elucidation. In this study, desorption electrospray ionization cyclic ion mobility mass spectrometry imaging (DESI-cIM-MSI) data from human renal cell carcinoma (RCC) tissues is used as a testbed to explore the extent to which CCS measurements enhance MSI lipid annotation confidence when combined with machine learning CCS predictions and SIRIUS analysis of MS^2^ data. Multipass IM experiments yielded excellent CCS accuracy (<0.4%) relative to database values for differential lipids found in RCC tissues, improving the filtering threshold used in previous CCS-based annotation workflows. High-accuracy multipass CCS measurements enabled the correct annotation of isobaric lipid database matches, even in the absence of MS^2^ data. Additionally, MS^2^ data from differential RCC features were uploaded to SIRIUS, and the predicted CCS values for SIRIUS candidates were compared to experimental CCS data to filter out unlikely candidates. Finally, CCS measurements contributed to the annotation of two spatially correlated unknown features, differential between tumor and control kidney tissues. Both features were assigned to rocuronium, a surgical muscle relaxant that had not been previously reported in MSI studies. Overall, these results underscore the potential of high-accuracy CCS values to enhance metabolite annotations in MSI-based spatial metabolomics.

## Graphical abstract

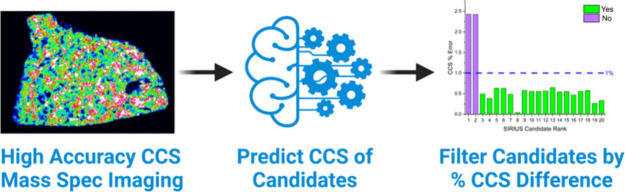

## Introduction

Nontargeted metabolomics studies aim to maximize annotation coverage;^1^ however, confidently annotating thousands of metabolites remains a significant challenge.^2^ Typically, fewer than 10% of features are successfully annotated, leaving the remaining unknown metabolites without plausible database matches.^3,4^ Without proper annotation, these metabolites offer no biological insights, yet they represent a largely untapped source of potentially valuable information. Further complicating these annotation challenges, acquiring MS^2^ data is typically constrained by low precursor ion abundance and ion coselection.^5,6^

Measurement of ion mobility (IM) collision cross sections (CCS), the two-dimensional average representations of molecular structure derived from IM drift times, is emerging as a robust approach to help illuminate the unannotated (or ″dark″) portion of the metabolome. These measurements are particularly useful in mass spectrometry imaging (MSI), which traditionally lacks front-end separations. IM platforms including drift tube ion mobility (DTIM) mass spectrometry (MS),^7,8^ trapped ion mobility spectrometry-time-of-flight (timsTOF)^9^ MS and cyclic ion mobility^10^ (cIM) MS have also been increasingly leveraged to resolve isobaric or interfering species in MSI by providing an additional separation dimension.^11^

CCS measurements derived from IM experiments have been successfully used to help annotate metabolites and lipids.^12–18^ A recent study, for example, combined liquid chromatography tandem mass spectrometry (LC-MS/MS) and LC-IM-MS to annotate differential metabolites using *de novo* molecular structure generation with SIRIUS 4 and CCS prediction with CCSP 2.0.^15,19^ SIRIUS constructs fragmentation trees from MS^2^ data to trace the progression of precursor ions to product ions,^20,21^ while CSI:FingerID (built into the SIRIUS suite) searches these molecular fingerprints against large structural databases to generate candidates.^22^ CCSP 2.0, a support vector machine-based algorithm, predicts CCS values by finding a combination of molecular descriptors that leads to the most accurate CCS predictions. By comparing predicted CCS values of SIRIUS candidates to experimental CCS data, an average of 28% of candidate structures with CCS differences exceeding ± 3% could be filtered.^15^ However, CCS filter thresholds could not be lowered due to the boundaries imposed by insufficient IM resolving power and CCS calibration errors.

cIM MS overcomes previous IM platform limitations by offering user-defined separation times that increase resolving power in proportion to the square root of the number of passes through the cIM cell.^10^ While earlier traveling wave IM (TWIM) platforms achieved a resolving power of R≈40 (t_d_/Δt_d_), a single pass through the cIM cell yields R≈65, and six passes results in R≈140.^10,23^ Six pass MSI experiments have demonstrated separation and imaging of isobaric lipids.^11^ Separating lipid isomers often requires resolving powers of 250 or greater,^24^ which can be achieved with cIM. IM resolution improvements have led to a better CCS calibration approach, recently proposed by Lin and Costello, which leverages the measurement of unperturbed drift times to achieve lower CCS errors.^25^ Such increased CCS accuracy allows for a reduction in the previous ± 3% threshold used for filtering candidate structures, enhancing both the filtering rate and the overall effectiveness of the workflow.

In this study, we leverage desorption electrospray ionization cyclic ion mobility mass spectrometry imaging (DESI-cIM-MSI) to study differential features observed in human clear cell renal cell carcinoma (ccRCC) tissues when compared against matched controls from the same patient. cIM CCS values for such differential features were determined using both the instrument’s default calibration method and the Lin and Costello method.^25^ Among 13 differential RCC lipids, for example, the average difference against the Baker and Siuzdak CCS database^26^ was 0.4% using the Lin and Costello method and 0.7% with the default method. We demonstrate that lowering the CCS difference threshold to ± 1% effectively filters out a larger number of potential SIRIUS candidates, thereby improving annotation confidence. Application of this workflow led to the annotation of two unknown differential features, later confirmed as originating from rocuronium, a surgical muscle relaxant not previously reported in spatial metabolomics studies. Among other differential features, triglycerides (TG) were found to be more abundant in tumor tissues, while coenzyme Q10 metabolites were more abundant in controls. To our knowledge, this is the first demonstration using cIM CCS values to enhance metabolite annotation in MSI studies.

## Methods and Materials

### Chemicals and Materials

Deionized water was used to ice-mount brain tissues to the microtome specimen holder. Kidney sections (20 μm) were collected onto Superfrost Plus microscope slides (Fisherbrand). The DESI solvent mixture was prepared using water (Fisher Optima, LC-MS grade), methanol (Fisher Optima, LC-MS grade), formic acid (Fisher Optima, LC-MS grade) and leucine-enkephalin (>95%, Sigma-Aldrich L9133). Major mix (Waters Corporation, Milford, MA) was used for CCS calibration.

### Kidney Tissue Samples

All patients provided informed consent on an Emory University IRB approved protocol (IRB #00055316). Patients at Emory University Hospital with a solid renal mass concerning RCC were identified prospectively. Histologic diagnosis of RCC was confirmed for each case. Excess tissue containing kidney tumor was obtained in the pathology lab, placed on ice, and transported to the processing lab. On ice, tissue was cut into pieces and placed in cold 2 mL cryovials then stored immediately in a −80 °C freezer. Frozen samples were transferred on dry ice to the Georgia Institute of Technology for MSI experiments.

### Mass Spectrometry Imaging and Instrumental Parameters

Slide-mounted tissues were placed in a desiccator 10 min prior to imaging. Imaging data were collected on a SELECT SERIES Cyclic IMS MS instrument (Waters Corporation, Milford, MA) equipped with a DESI-XS ion source (Waters Corporation, Milford, MA). Four tumor kidney sections and four matched control kidney sections were imaged in positive ion mode using one pass through the cIM cell. One control kidney section was used to collect positive ion mode data at various IM passes. CCS values from this data set were calculated with the Lin and Costello multipass calibration approach.^25^ DESI images were collected from *m*/*z* 50 to 1200 with a 50 μm raster width at a scan rate of 250 μm sec^–1^ (0.2 s per pixel). The DESI solvent mixture (methanol/water, 98:2, 0.1% formic acid with leucine-enkephalin, 200 pg ul^–1^) was electrosprayed at 0.7 kV with a 2 μL min^–1^ flow rate using a nanoflow pump (M-Class uBSM, Waters). Additional MS settings are provided as a supplementary.txt file.

### Image Processing and Data Analysis

DESI-cIM MS images were processed with the Waters HDI 1.7 software with leucine-enkephalin as the lock mass (*m*/*z* 556.2771), 0.25 Da lock mass tolerance, 500 min signal intensity, 30 min sample frequency, 10 s sample duration, 0.05 Da *m*/*z* window, 40,000 MS resolution, 1 bin start drift, 200 bins stop drift, 2 bins drift window and 4 bins HD min peak width. Averaged *m*/*z* and drift time (t_d_) lists (target lists) containing the 1000 most abundant features for positive ion mode single-pass data were built in HDI using all eight kidney tissue samples, a 0.03 Da *m*/*z* tolerance and 2 bin drift tolerance. Metabolite abundance data were extracted from whole kidney sections using the polygon tool in HDI and exported for statistical analysis. Nonendogenous background ions were manually removed. Features were further filtered with a 1.5-fold change and a 0.1 *p*-value threshold.

### Feature Annotation

MSI putative feature annotation was based on matches to *m*/*z* values from the averaged target lists using LIPID MAPS^27^ (https://www.lipidmaps.org) and HMDB^28^ (https://hmdb.ca). The *m*/*z* tolerances used for LIPID MAPS and HMDB were ± 0.005 Da and ± 5 ppm, respectively. The adduct ions searched for were [M+H]^+^, [M+Na]^+^, [M+K]^+^ and [M+2H]^2+^. Liquid chromatography mass spectrometry experiments, detailed later, were conducted to collect MS^2^ data on differential features to confirm putative MS^1^ database matches.

### CCS Calculations

CCS values were calculated using the instrument default approach and the Lin and Costello approach.^25^ For the default approach, CCS was calibrated using the manual calibration option with major mix. The “mob_cal.xlsx” file contained within the instrument data folders relates corrected CCS values and corrected drift time values for major mix analytes. A linear fit (y = mx + b) was created between these values to extract m and b used in eq 3. First, the drift time in bins (t_d(bins)_) from the differential RCC features was converted to drift time (t_d(ms)_) using eq 1. V_ADC_ was obtained from the “_extern.inf” file within the data folder and is labeled as “ADC Pusher Period (us)”. The drift time (t_d(ms)_) was then converted to corrected drift time (t_c_*) using eq 2. C was obtained from the “_extern.inf” file within the data folder and is labeled as “Transfer.EDCCoefficientHigh.Setting”. In this equation, *m*/*z* is the mass to charge ratio of the ion. The corrected drift time (t_c_*) was then converted to corrected CCS (Ω_*c*_) using eq 3. Finally, the corrected CCS (Ω_*c*_) was converted to CCS (Ω) using eq 4, where *z* is the charge of the ion, μ is the reduced mass, calculated from 1/μ = 1/*m*_ion_ + 1/*m*_gas_, and *m*_gas_ is the mass of the drift gas used (N_2_ in this case).

$$t_{d\left(\mathrm{ms}\right)}=\frac{t_{d\left(\mathrm{bins}\right)}v_{\mathrm{ADC}}}{1000}$$ 1

2 $${t_{c}}^{*}=t_{d\left(\mathrm{ms}\right)}-C\sqrt{m/z}$$

3 $$\Omega_{c}={{\mathrm{mt}}_{c}}^{*}+b$$

$$\Omega^{\prime }={\Omega_{c}}^{*}z\sqrt{1/\mu }$$ 4

The Lin and Costello multipass approach was implemented to account for fluctuations in periodic drift time due to the electric field switching utilized to eject ions from the cIM separator after mobility separation, which introduces a perturbation to the electric potential within the cyclic path for a given separation time (*t*_*s*_).^25^ Briefly, arrival times for calibrants in major mix were determined through Gaussian fitting. Linear fitting of eq 5 (below) yielded *t*_*pp*_, the perturbed periodic drift time (time spent under the perturbed field condition after *t*_*s*_). In this equation, *t*_*nd*_ is the total ion drift time in the cIM separator, *n* is the number of passes through the separator, and *v*_*u*_ and *v*_*p*_ are the velocities under unperturbed and perturbed field conditions, respectively. The *t*_*pp*_ of each calibrant along with literature drift tube ion mobility CCS values^29^ were used to create a calibration curve via a power law function. Refined CCS values for kidney lipids were determined from the multipass calibration curve using their *t*_*pp*_ values.

5 $$\frac{\left(t_{nd}-t_{s}\right)}{n}=t_{pp}-\frac{v_{u}}{v_{p}}\frac{t_{s}}{n}$$

### Liquid Chromatography Mass Spectrometry

Reversed phase ultrahigh-performance liquid chromatography tandem mass spectrometry was collected on a mixture of control and tumor kidney extracts. An inclusion list targeting all differential RCC features from the cIM imaging data was used to collect MS^2^ data. The reversed phase LC-MS/MS method used has been previously described in detail.^30^ High-energy collision dissociation (HCD) data were collected in an Orbitrap Exploris 240 mass spectrometer (ThermoFisher Scientific). Chromatography was conducted with a Thermo Accucore C_30_, 150 × 2.1 mm, 2.6 μm particle size column. Mobile phase A was 10 mM ammonium acetate 0.1% formic acid in 40:60 v/v water/acetonitrile. Mobile phase B was 10 mM ammonium acetate 0.1% formic acid in 90:10 v/v isopropanol/acetonitrile.

### SIRIUS 6

LC-MS/MS data was converted from .raw to .mzML using MSConvert and uploaded to SIRIUS 6. The SIRIUS parameters used for computation of structures were *de novo* and bottom up molecular formula generation, 5 ppm MS^2^ accuracy, [M + H]^+^, [M + Na]^+^, [M+K]^+^ adducts, H,C,N,O,P, S elements for *de novo*, all databases and approximate confidence mode. *De novo* structures were not considered except for unknown identification. For each lipid entry in SIRIUS, SMILES of the top 20 candidates were extracted and used to predict their CCS using CCSP 2.0.^19^ Each candidate structure was examined to determine whether the lipid sum composition matched the MS^1^ database entry.

### CCS Prediction 2.0 (CCSP 2.0)

Details of the machine learning-based CCS prediction tool, CCSP, have been provided elsewhere.^15,19^ Data from the Unified CCS Compendium^16^ were used to train and validate CCSP with 70% of the data allocated to training and the remaining 30% used for validation. The training set input was composed of an ID, a CCS value, and a molecular structure input such as SMILES or InChI. The target set input is an ID and a molecular structure input. Predictions were carried out separately for [M+H]^+^ and [M+Na]^+^ ions. CCSP was trained with each of these ion types found in the Unified CCS Compendium.

## Results and Discussion

### Differential RCC Features

Human tumor kidney tissue (*n* = 4) and matched normal kidney tissues (*n* = 4) were imaged in positive ion mode with one pass through the cIM cell (Figure 1A). In all cases, the tumors were identified as ccRCC, the most common type of kidney cancer, known for its imbalance in lipid metabolism^31^ and enhanced lipogenesis that is responsible for its histological appearance. A feature list containing the 1000 most abundant ions was generated from all eight samples. After aligning *m*/*z* and drift time and removing background ions, 216 features remained. To refine the list, we applied a 1.5-fold-change (FC) and 0.1 *p*-value cutoff, reducing the feature set to 179. Given the small sample size, we used a 0.1 *p*-value threshold to ensure sufficient features for demonstrating the proposed annotation workflow rather than drawing biological conclusions about RCC.

**Figure 1:**
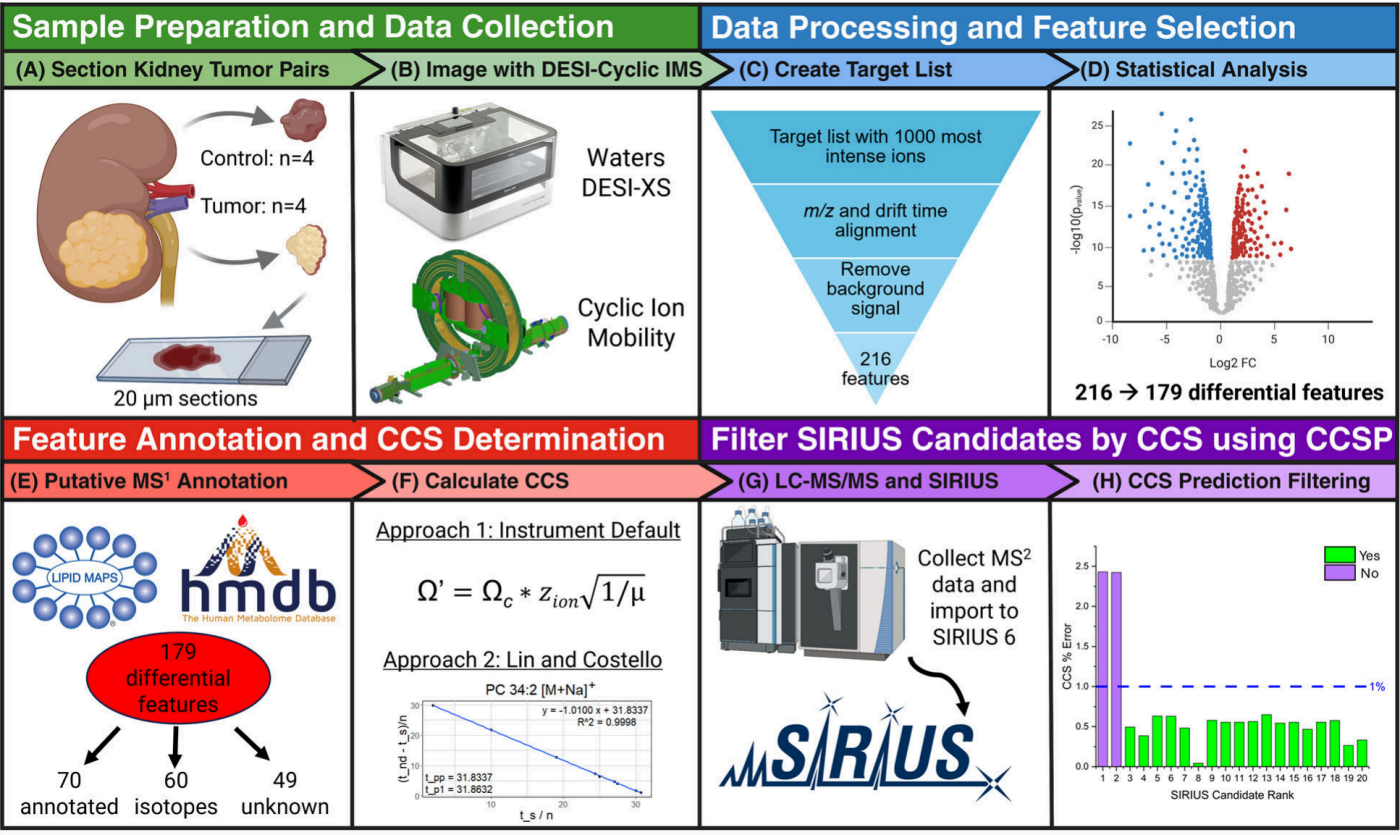
Experimental workflow. (A) Human tumor kidney tissue (*n* = 4) and matched normal kidney tissues (*n* = 4) were sectioned at 20 μm. (B) Imaging data were collected on a Waters Select Series Cyclic IMS platform equipped with a Waters DESI-XS ion source in positive ion mode using one pass through the cyclic ion mobility cell for all samples. (C) MS images were processed with Waters HDI 1.7 software. Averaged *m*/*z* and *t*_d_ lists containing the 1000 most abundant features were built using all samples. Nonendogenous background ions were manually removed from the list. Metabolite abundance data were extracted from whole kidney sections and exported for statistical analysis. (D) Feature lists were further filtered with a 1.5-fold change and a 0.1 *p*-value threshold, yielding 179 differential RCC features. (E) Putative feature annotation was conducted using LIPID MAPS and HMDB, producing 70 tentative identities, 60 isotopic species, and 49 unknowns. (F) CCS values were measured using two different calibration approaches outlined in Figure 3. (G) LC-MS/MS data were collected on a pooled sample of control and tumor kidney extracts, targeting all differential RCC features observed in the DESI cIM-MS imaging data set. LC-MS/MS data was uploaded to SIRIUS 6 and (H) CCSP was used to predict CCS values for the top SIRIUS candidates of each differential lipid and compared to experimental CCS values to filter out false positive matches.

Of the 179 differential features, 70 had putative database matches to LIPID MAPS and HMDB, all with mass error ± 5 ppm (Table S1). Many of these annotations were later confirmed with LC-MS/MS analysis. An additional 60 features were tentatively identified as isotopic species (based on drift time, abundance ratio and *m*/*z*), while the remaining 49 features had no database matches and were classified as unknowns. A volcano plot (0.05 *p*-value, fold change >2) was generated to highlight the most significant metabolite changes between tumor and control kidney tissues (Figure S1). Ion images of a tumor-control pair are shown for two of these metabolites: TG(58:5) [M + Na]^+^ which had the most significant *p*-value in tumor tissues, and coenzyme Q10 [M+K]^+^ (Figure 2), which exhibited the largest fold change in the control tissues. Additionally, two unknown metabolites at *m*/*z* 529.3989 and 265.2030 were more abundant in control tissues, as discussed later in the manuscript.

**Figure 2:**
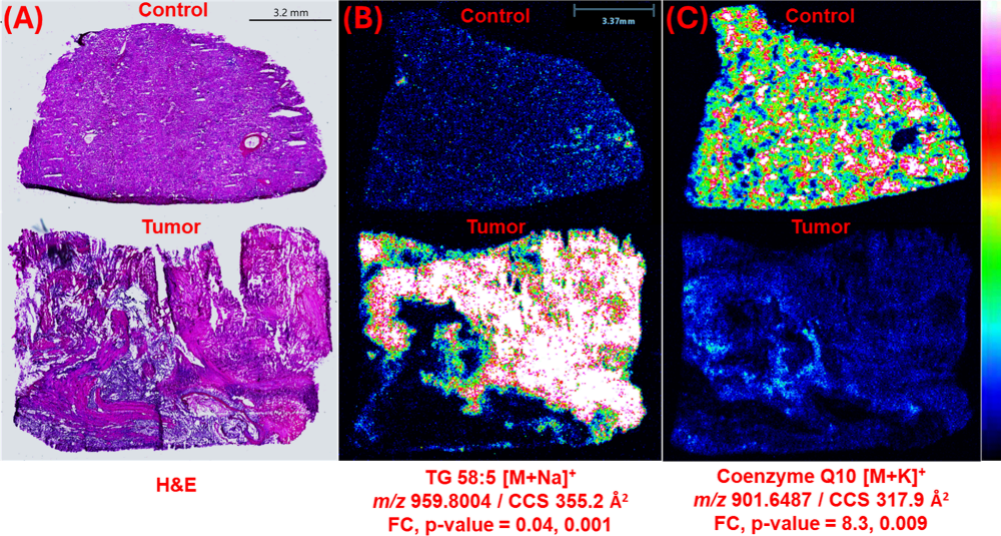
Differential renal cell carcinoma metabolites. (A) H&E image of one control/tumor kidney tissue pair. (B, C) Ion images for the same kidney samples, showing two highly differential metabolites. The TG(58:5) [M + Na]^+^ ion was more abundant in tumors with an FC (control/tumor) of 0.04 and *p*-value of 0.001, whereas the coenzyme Q10 [M + K]^+^ ion was more abundant in control tissues with an FC of 8.3 and *p*-value of 0.009.

### CCS Accuracy

CCS calibration using the instrument default approach and the Lin and Costello^25^ multipass approach are outlined in Figures 3A and 3B, respectively. Detailed descriptions of both methods are provided in the Methods section. The instrument default approach is based on a linear relationship between CCS and time-corrected drift time (Figure 3A), which deviates from the more commonly accepted power function-based calibration models. Power function models were explored, but resulted in reduced CCS accuracy for this data set, therefore we followed the linear calibration model. The CCS values resulting from these approaches were compared against the Baker/Siuzdak METLIN CCS database^26^ (Figure 3C) and the McLean CCS Compendium^16^ (Figure 3D). The percent CCS error between experimental and database values was calculated for the 13 differential lipids found in the METLIN CCS database and the 5 differential lipids found in the CCS Compendium. For lipids in the METLIN CCS database, the average CCS error was 0.7% using the instrument default and 0.4% using the Lin and Costello approach. Similarly, for lipids in the CCS Compendium, the average CCS error was 3.6% and 2.4% using the instrument default and Lin and Costello approach, respectively. These results demonstrated that the Lin and Costello multipass calibration approach provided more accurate CCS values than the default approach, regardless of the database used. In general, it was observed that the experimental CCS values aligned better with the METLIN CCS database than with the CCS Compendium. This discrepancy may be attributed to the METLIN CCS Database being acquired with TIMS-MS, which like the TWIM cIM method used in this study, requires calibration with standards. In contrast, the CCS Compendium was built using DTIM data, whose values are directly derived with the Mason-Schamp equation. Our findings suggest that TWIM-derived lipids CCS values are more consistent with TIMS measurements than with DTIM values. A study comparing CCS values derived from DTIM, TIMS and TWIM found that pairwise differences between TIMS and DTIM were slightly larger than those between TIMS and TWIM, or between TWIM and DTIM.^32^ The same study demonstrated that the median% CCS deviation was lower from TIMS versus TWIM as compared to TIMS versus DTIM for both positive and negative mode,^32^ supporting our findings here.

**Figure 3:**
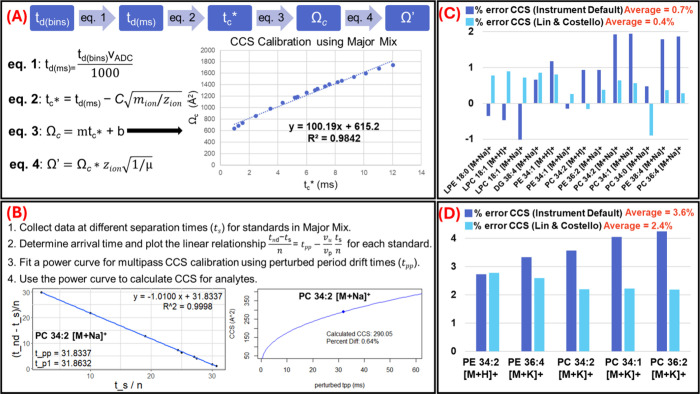
CCS calibration approaches employed and comparison to database values. (A) Instrument default calibration approach. (B) Lin and Costello approach.^25^ See Methods section for details. (C) Comparison of CCS calculation approaches to differential lipids found in the Baker/Siuzdak METLIN CCS Database.^26^ (D) Comparison of both CCS calibration approaches to differential lipids found in the McLean CCS Compendium.^16^ Overall, the Lin and Costello calibration led to more accurate CCS values. In general, these values aligned better with the METLIN CCS database reference values, rather than those in the CCS Compendium.

### Filtering Isobaric MS^1^ Lipid Matches by CCS

Collecting high quality MS^2^ data during MSI experiments is challenging for several reasons. In many workflows, MS^2^ is collected one metabolite at a time, making it a time-consuming venture for nontargeted experiments with hundreds of differential analytes. Sensitivity is another major limitation, as metabolite signals are generally much weaker in MSI than in LC-MS/MS, due to the spatially resolved nature of the experiment, further complicating MS^2^ spectral collection. Additionally, precursor ion isolation decreases the overall signal intensity of a precursor ion, and often undesired species are co-selected due to a lack of front-end separation, leading to chimeric spectra.^33^ All these limitations make collecting MS^2^ data particularly difficult for low abundance metabolites. One common answer to these issues is to conduct LC-MS/MS experiments on tissue extracts, but matching spectral data collected using different analytical techniques presents additional challenges.

CCS measurements offer an alternative means of annotating metabolites, especially when MS^2^ data is difficult to acquire. This approach is suitable for imaging platforms equipped with IM capabilities that can yield highly accurate CCS values. To evaluate the effectiveness of CCS in differentiating structurally similar compounds, CCS values were machine learning-predicted for features that produced two plausible isobaric lipid database matches. These predicted values were then compared to the features’ experimental CCS calculated with the Lin and Costello multipass approach (Figure 4). Since numerous lipid isomers exist for a given lipid sum composition, the structures used for CCS prediction with CCSP were selected based on commonly observed chain lengths such as 16:0, 16:1, 18:0, and 18:1. Five isobar pairs were evaluated using this approach, with one isobar being an [M+Na]^+^ ion and the second isobar being an [M+H]^+^ ion. In each case, the percent difference between predicted and experimental CCS was lower for the [M+Na]^+^ isobar than for the [M+H]^+^ isobar. Subsequent LC-MS/MS analysis confirmed that the correct identity for each of these MSI features was the [M+Na]^+^ isobar, demonstrating that high accuracy CCS values are able to distinguish structurally similar compounds. Importantly, this level of discrimination is unfeasible with lower accuracy CCS values as both isobaric species would fall within higher error margins. This experiment highlights the potential for cIM CCS measurements to enhance analyte identification in the absence of high quality MS^2^ data, offering a powerful tool for metabolite annotation in spatial metabolomics.

**Figure 4:**
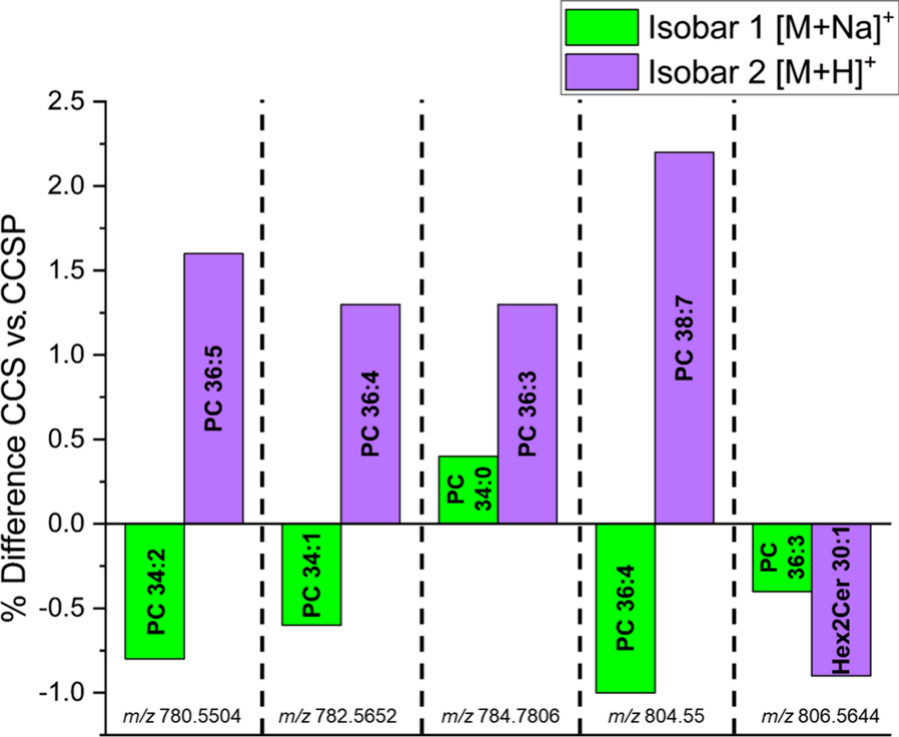
Filtering isobaric MS^1^ lipid matches by CCS. LIPID MAPS produced two plausible isobaric lipid matches for certain *m*/*z*, with one species being [M+H]^+^ and the other being [M+Na]^+^. The CCS value of each lipid was predicted with CCSP 2.0 and compared to the experimental CCS value. In all cases, the predicted CCS value of the [M+Na]^+^ isobar was in better agreement with the experimental CCS value than that of the [M+H]^+^ isobar. Subsequent LC-MS/MS analysis confirmed that the correct identity of each feature was the [M+Na]^+^ isobar selected by CCS filtering.

### Filtering SIRIUS Candidates by CCS

To further demonstrate the value of high accuracy CCS measurements in MSI lipid annotation, LC-MS/MS data was uploaded to SIRIUS and candidates filtered by comparing their predicted CCS values to experimental CCS values (Figure 5 and Figure S2). LC-MS/MS were collected on all differential RCC features from the imaging experiments and uploaded to SIRIUS 6. CCS values were predicted for the top 20 SIRIUS candidates for each lipid, and each candidate structure was assessed to determine whether the lipid sum composition matched the MS^1^ database putative annotation. Given the average 0.4% CCS error achieved for the 13 lipids relative to database values (Figure 3), a ±1% CCS threshold was applied to discard SIRIUS candidates. Figure 5 highlights four different examples where CCS values were calculated using the Lin and Costello multipass approach. For the PE(34:1) [M+H]^+^ ion the top two candidates did not match the lipid sum composition and had a 2.4% CCS difference whereas the remaining 18 matching candidates had an average 0.5% difference (Figure 5A). Similarly, the top three incorrect candidates for the PE(34:2) [M + H]^+^ ion exhibited nearly a 4% difference (Figure 5B). Across all six nonmatching candidates, the average CCS difference was 2.2%, noticeably higher than the 0.7% difference observed for the remaining matching candidates. For the PC(34:1) [M+K]^+^ ion, 13 candidates were incorrectly predicted as [M+H]^+^ species (Figure 5C), resulting in a significantly higher average CCS difference of 5.3%. In the case of the PC(34:2) [M+Na]^+^ ion, the only incorrect candidate had a 3.1% difference, while the remaining 19 correct candidates had an averaged 0.4% CCS difference (Figure 5D). Two additional examples using the instrument default CCS calibration approach for the PC(34:2) [M+H]^+^ ion and the PC(36:3) [M+Na]^+^ ion are shown in Figure S2. Most incorrect SIRIUS candidates contained cyclopropane or hydroxyl modifications in their fatty acyl chains, which led to deviations in their predicted CCS, allowing them to be filtered out. However, lipid double bond and chain length isomers generally produced predicted CCS within ± 0.5% of each other and could not be differentiated by CCS, emphasizing the need for even more accurate IM measurements. In summary, the enhanced accuracy of CCS values provided by the higher IM resolution in cIM, in combination with the more accurate multipass CCS calibration approach enabled the previous ± 3% difference between experimental and predicted CCS values to be successfully reduced to ± 1%. This improvement enhanced the utility of CCS as a confirmation metric in lipid annotation.

**Figure 5:**
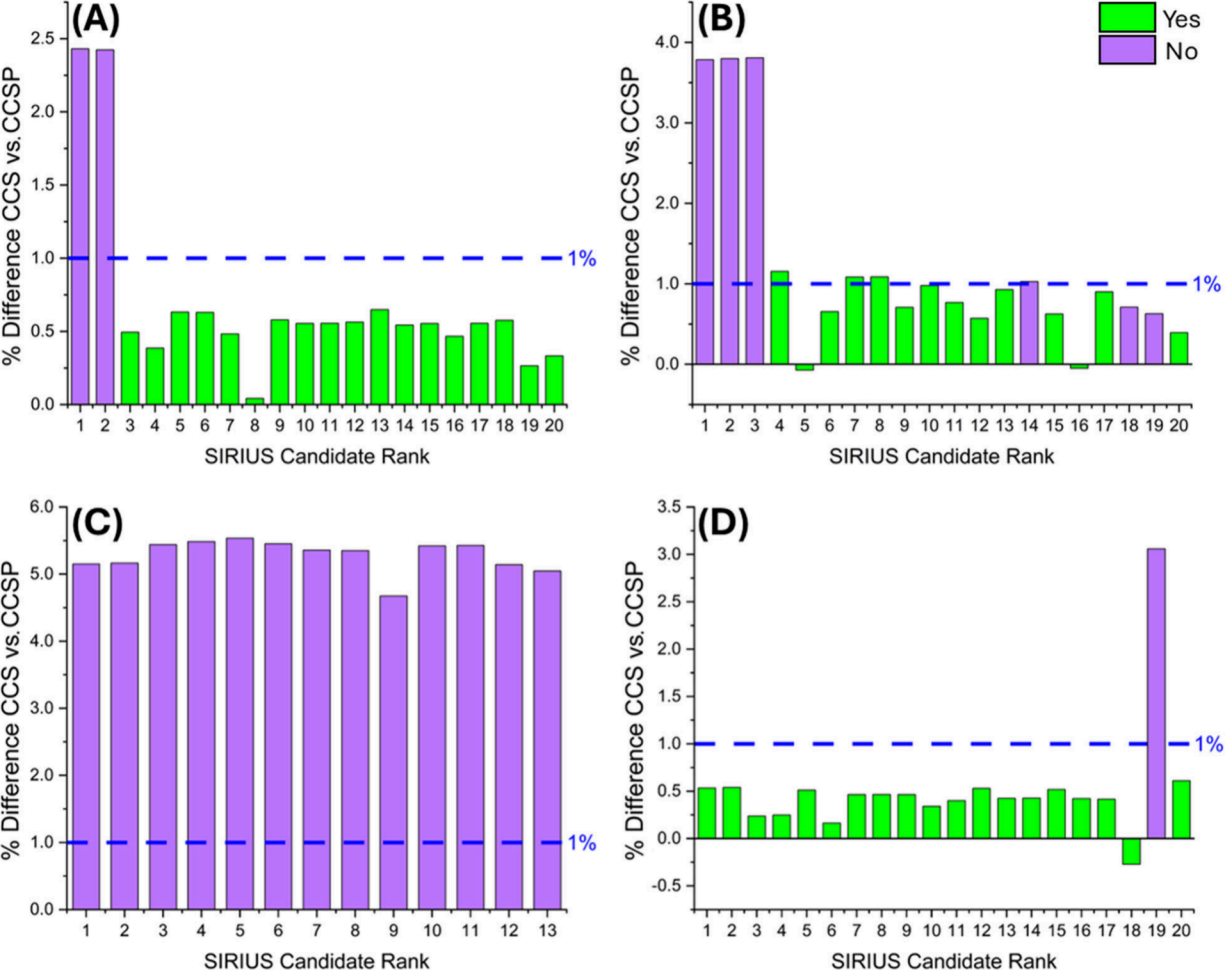
Filtering SIRIUS candidates by CCS. LC-MS/MS data were collected on all differential RCC features identified in the imaging data set and uploaded to SIRIUS 6. CCS prediction was performed for the top 20 SIRIUS candidates of each lipid. Each candidate structure was assessed to determine whether its lipid sum composition matched the MS^1^ database putative annotation, green indicating a match and purple indicating no match. Machine learning-predicted CCS values were compared to experimental CCS values calculated from the Lin and Costello calibration approach. A 1% CCS difference threshold was applied to filter out unlikely structures. The majority of retained structures corresponded to candidates that matched the putative MS^1^ lipid sum composition. (A) PE(34:1) [M+H]^+^. (B) PE(34:2) [M+H]^+^. (C) PC(34:1) [M+K]^+^. (D) PC(34:2) [M+Na]^+^.

### Leveraging CCS to Pursue Unknown Metabolite Annotation

Two unknown species at *m*/*z* 529.3989 and 265.2030 (Figure 6) were significantly more abundant in control kidney tissues relative to tumors (FC = 0.18, *p*-value = 0.018 and FC = 0.20, *p*-value = 0.028, respectively) but lacked clear database matches to endogenous lipids or metabolites. The feature at *m*/*z* 265.2030 exhibited a relatively low drift time, suggesting it was doubly charged. This was confirmed by the presence of the isotope at *m*/*z* 265.7046. Additionally, *m*/*z* 529.3989 and 265.2030 showed highly correlated spatial distributions, leading to calculations confirming *m*/*z* 265.2030 was indeed the doubly charged ion of 529.3989. However, metabolites at this *m*/*z* do not typically form doubly charged species, adding to the peculiar nature of these unknown features. LC-MS/MS data was acquired on *m*/*z* 529.3989 and uploaded to SIRIUS, which produced 100 *de novo* structures and 8 database matches. CCS values were predicted for all of these species. Applying a ± 1% difference between predicted and experimental CCS values as a cutoff, 78 out of the 108 candidates were eliminated, leaving 30 candidates for further investigation (Figure 6C). Reducing the cutoff to 0.5% would have allowed 91 candidates to be discarded. However, none of the remaining *de novo* structures made sense, biologically.

**Figure 6:**
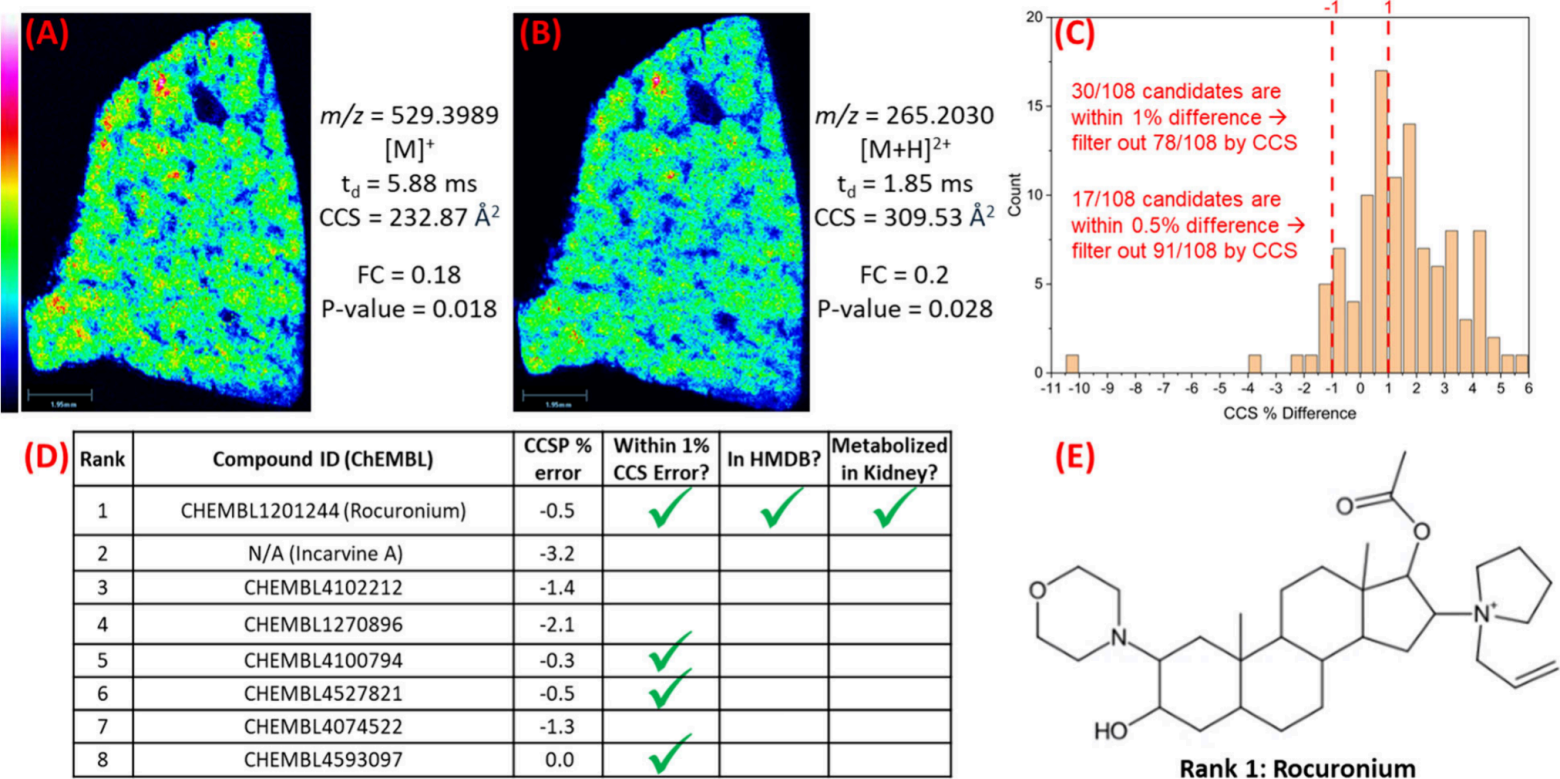
Leveraging CCS to pursue unknown metabolites. (A) Ion image of *m*/*z* 529.3989 and (B) ion image of *m*/*z* 265.2030 in a control kidney. (C) LC-MS/MS data from *m*/*z* 529.3989 was uploaded to SIRIUS, which produced 100 *de novo* structures and 8 database matches. CCS values of these structures were predicted and compared to the experimental CCS. Applying a ±1% CCS difference as a cutoff, 78 out of 108 candidates were eliminated, leaving 30 candidates for further investigation. Reducing the cutoff to 0.5% would have allowed 91 candidates to be discarded. (D) Table comparing the 8 database matches produced by SIRIUS. Four out of eight candidates were within 1% CCS difference, but only the top candidate was in HMDB and was reported to be metabolized in the kidney. (E) Structure of the top SIRIUS candidate, rocuronium. Figure S3 contains the structures of all 8 database candidates.

The 8 database candidates (structures provided in Figure S3) were carefully re-examined. Only the top ranked candidate was found in HMDB, while others were found only in natural product databases or PubChem (Figure 6D). The top ranked candidate was rocuronium, a drug used for endotracheal intubation and skeletal muscle relaxation during surgery (Figure 6E). A literature search confirmed that this drug is partially metabolized in the kidney^34^ and is detected as the [M]^+^ and [M+H]^2+^ ions.^35^ The mass error for *m*/*z* 529.3989 and 265.2030 was −3.1 and −4.4 ppm, respectively, further supporting rocuronium as the correct annotation. Additionally, the difference in predicted CCS and experimental CCS of the *m*/*z* 529.3989 rocuronium ion was only 0.5%, providing additional confirmation. Literature characterizing the fragmentation of rocuronium showed that the dominant fragment ion was at *m*/*z* 487.3525, resulting from a propenyl loss, followed by loss of acetate at *m*/*z* 427.3315 and loss of pyrrolidine at *m*/*z* 340.2633.^36^ In the LC-MS/MS data collected here, the most abundant fragment of *m/z* 529.3997 (error = −1.6 ppm) was *m/*z 487.3533 (error = −0.6 ppm) corresponding to the propenyl loss (Figure S4). Additionally, fragments from the following acetate and pyrrolidine loss were also detected (error = −2.7 and −0.7 ppm, respectively), along with several other characteristic rocuronium fragments described in the literature (Figure S4).^36^ Collectively, these chemical data confirmed the annotation of rocuronium. Subsequent examination of patient records revealed that all patients had received rocuronium on the day of surgery, though this information was unknown during the annotation process. The strong rocuronium signal can be attributed to the quaternary ammonium moiety in its structure, which carries a permanent positive charge. Ion images of rocuronium in all tumor-control pairs are provided in Figure S5. The images indicate that rocuronium accumulates in the control kidney samples, but not in the tumor samples. To our knowledge this is the first time rocuronium has been reported in an MSI experiment. Furthermore, this is the first demonstration of how CCS values derived from MSI can facilitate the annotation of unknowns or unexpected database matches. This finding underscores the unique advantages provided by cIM in unknown annotation in MSI.

## Conclusions

This study demonstrates the value of combining CCS measurements and candidate structure CCS predictions to enhance metabolite identification confidence in nontargeted spatial metabolomics via MSI. By leveraging the high ion mobility resolution of cyclic IMS along with a new multipass CCS calibration approach reported by Lin and Costello, an average CCS error of only 0.4% was achieved for 13 differential RCC lipids, relative to database values. These high-accuracy CCS values allowed for the accurate differentiation of isobaric lipid database matches, showing that CCS can serve as an additional metric for lipid annotation even in the absence of MS^2^ data. Such a distinction would have been impossible with lower accuracy CCS values, where both isobars’ CCS would fall within the accepted error range, making them indistinguishable. To further harness CCS values for metabolite identity confirmation, SIRIUS candidates were filtered based on a ± 1% difference between their predicted and experimental CCS values, a refined threshold reduced from the ± 3% used in previous studies, thereby enabling more effective candidate filtering. Finally, CCS values were used to identify two unknown features as rocuronium, a surgical muscle relaxant previously unreported in MSI studies. These findings underscore the potential of high-accuracy CCS measurements as a powerful tool for enhancing metabolite identification confidence.

## Supplementary Material





## Notes

Figure S1 shows a volcano plot of differential renal cell carcinoma metabolites; Figure S2 provides additional examples of filtering SIRIUS candidates by CCS; Figure S3 provides the structures for all SIRIUS database candidates of the unknown at *m*/*z* 529.3989; Figure S4 shows the rocuronium fragmentation pattern for LC-MS/MS data.; Figure S5 shows ion images for rocuronium in all kidney samples; Table S1 contains data on differential RCC analytes including their identity, mass error, drift time, fold-change, and *p*-value (PDF)
Additional Cyclic IMS parameters (TXT)
