# Supplementary material for: A Spatial Metabolomics Annotation Workflow Leveraging Cyclic Ion Mobility and Machine Learning-Predicted Collision Cross Sections
Source: J Am Soc Mass Spectrom. 2025 May 21;36(6):1386–94. doi: 10.1021/jasms.5c00090 (PMC12142677; doi:10.1021/jasms.5c00090)
Supplement: Supplementary file 1 [file js5c00090_si_001.pdf]

Supplemental Information for:

A Spatial Metabolomics Annotation Workflow Leveraging Cyclic Ion Mobility and Machine Learning-Predicted Collision Cross Sections.

Dmitry Leontyev[1], Eric C. Gier[1], Viraj A. Master[2,3], Rebecca S. Arnold[3], John A. Petros[3], Facundo M. Fernández[1,4]

[1]School of Chemistry and Biochemistry, Georgia Institute of Technology, Atlanta, GA 30332 (USA).

[2]Winship Cancer Institute, Emory University, Atlanta, GA 30322 (USA).

[3]Emory School of Medicine, Department of Urology, Atlanta, GA 30342 (USA).

[4]Parker H. Petit Institute for Bioengineering and Bioscience, Atlanta, GA 30332 (USA).

*Email: facundo.fernandez@chemistry.gatech.edu

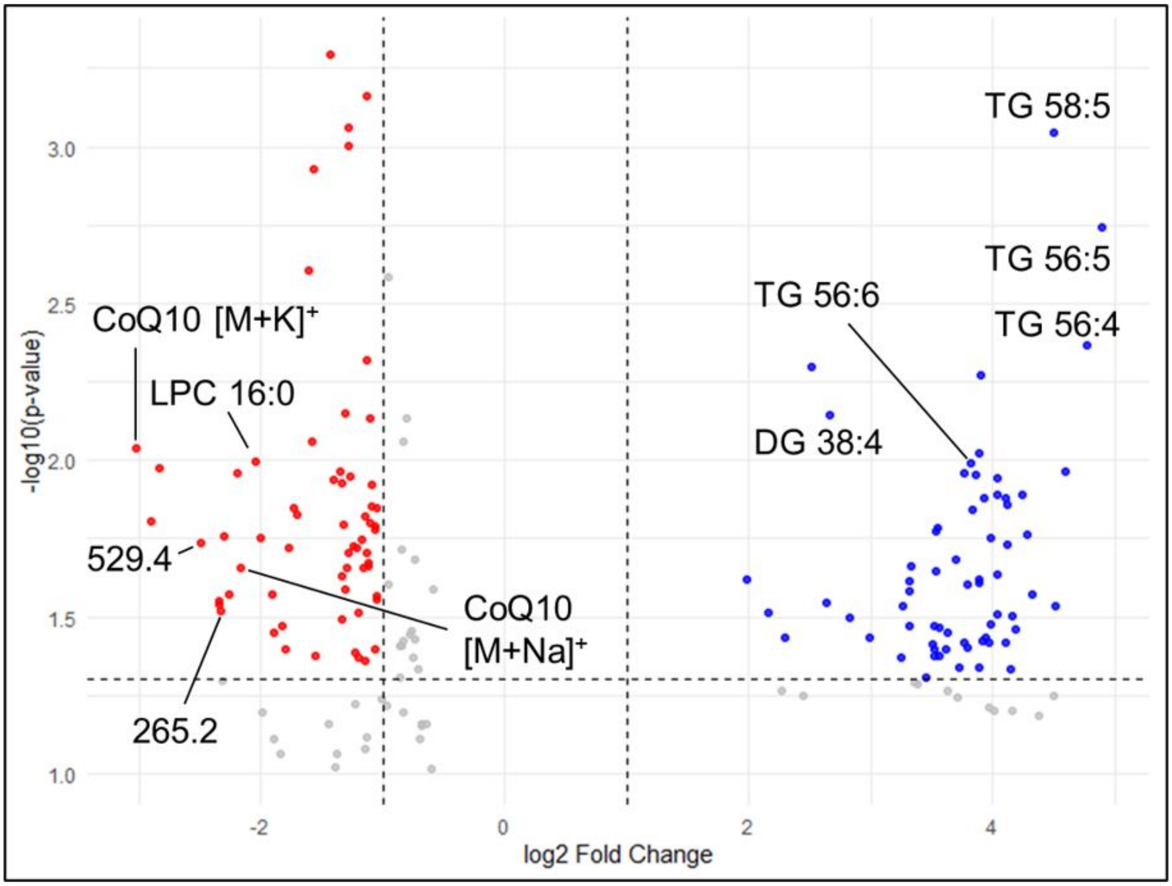

Figure S1. Differential Renal Cell Carcinoma Metabolites. Volcano plot (0.05 p-value, fold change > 2) for all 216 features highlighting differential metabolites, including two salient unknowns labeled with their *m/z* values.

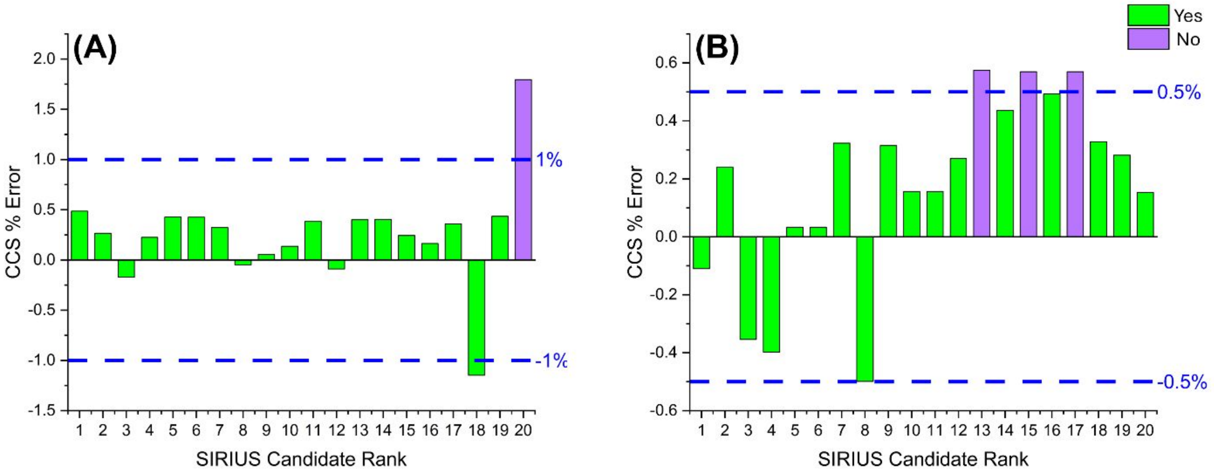

Figure S2. Filtering SIRIUS Candidates by CCS values derived from the Default Instrument Calibration Approach. LC-MS/MS data were collected on all differential RCC features selected from the imaging dataset. This data was uploaded to SIRIUS 6 and CCS prediction was performed on the top 20 SIRIUS candidates for each lipid. Each candidate structure was examined to determine whether the lipid sum composition matched the MS^1 putative database annotation (green indicating yes and purple indicating no). The predicted CCS values were compared to the experimental CCS calculated with the default instrument calibration approach. **(A)** PC(34:2) $[M+H]^+$. **B)** PC(36:3) $[M+Na]^+$.

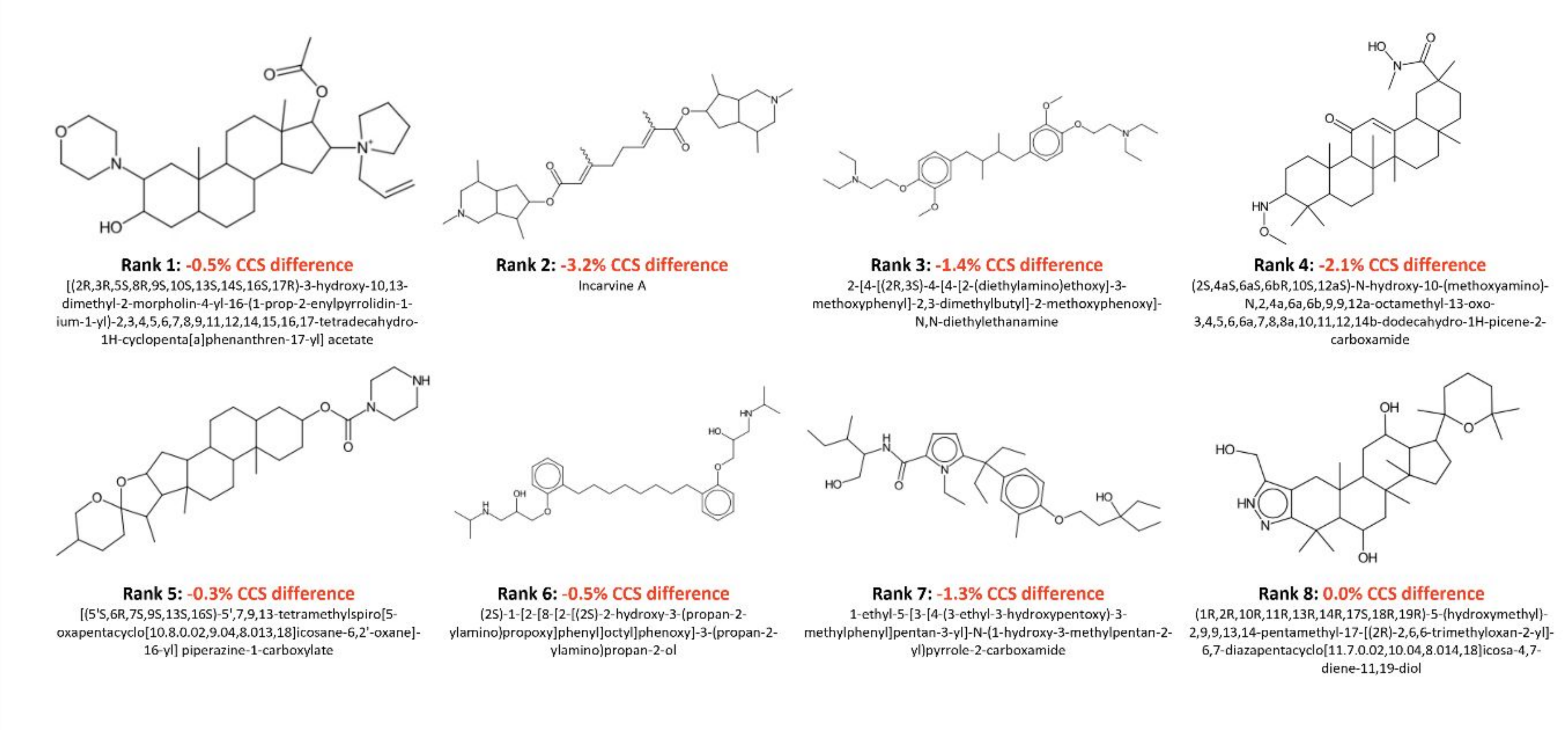

Figure S3. SIRIUS Candidate Structures for Unknown Metabolite at *m/z* 529.3989. LC-MS/MS data for *m/z* 529.3989 was uploaded to SIRIUS, which produced 100 *de novo* structures and 8 database matches. Shown here are the structures of the 8 database matches and the difference in CCS between experimental and predicted values.

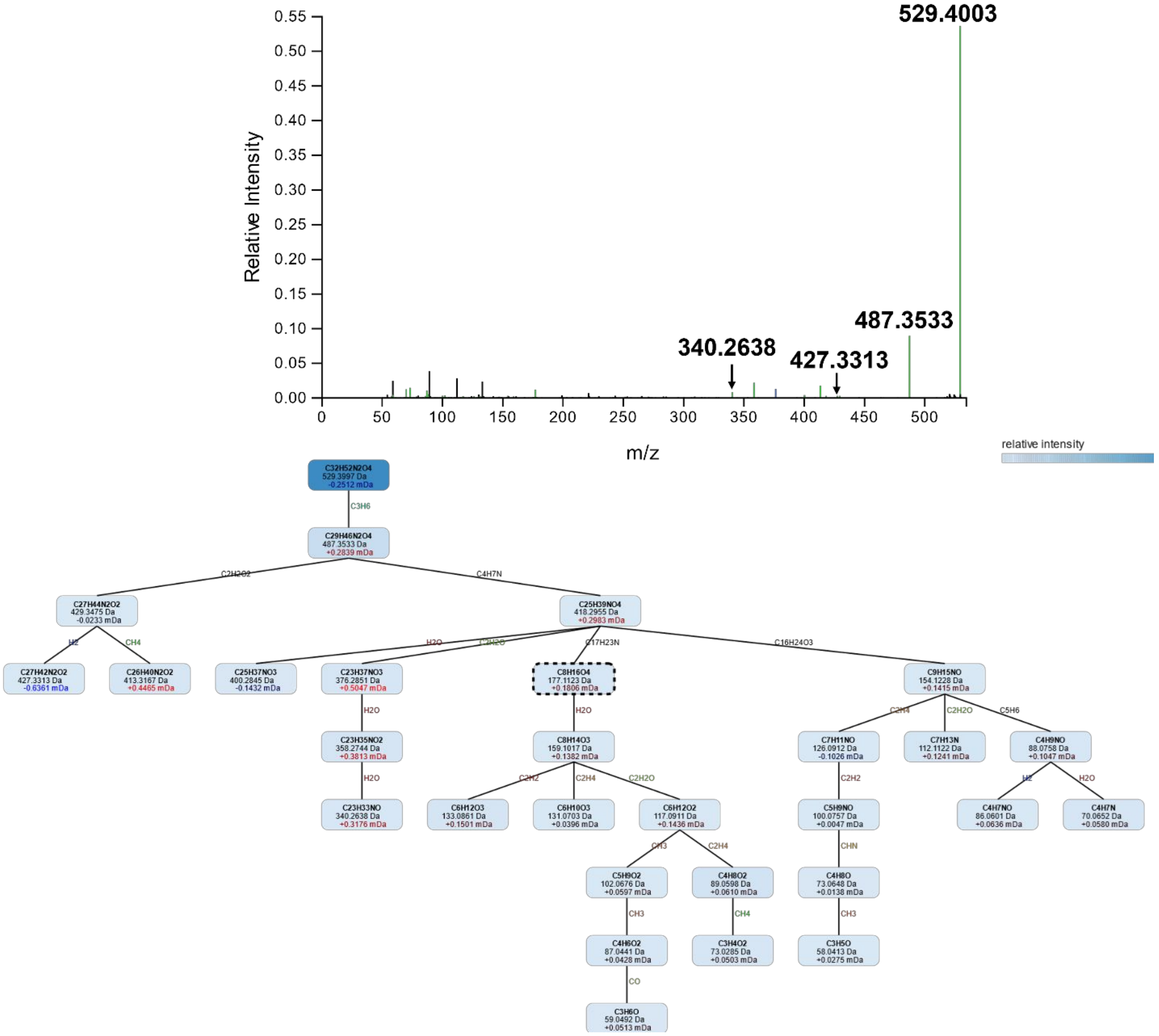

Figure S4. Rocuronium Fragmentation from LC-MS/MS Data and SIRIUS Fragmentation Tree. The MS/MS spectra is labeled with the major rocuronium fragments. The ion at *m/z* 487.3533 results from a propenyl loss, followed by loss of acetate at *m/z* 427.3313 and loss of pyrrolidine at *m/z* 340.2638. The fragmentation tree outlines the progression of precursor ion to product ions for rocuronium and includes a number of additional characteristic fragments.

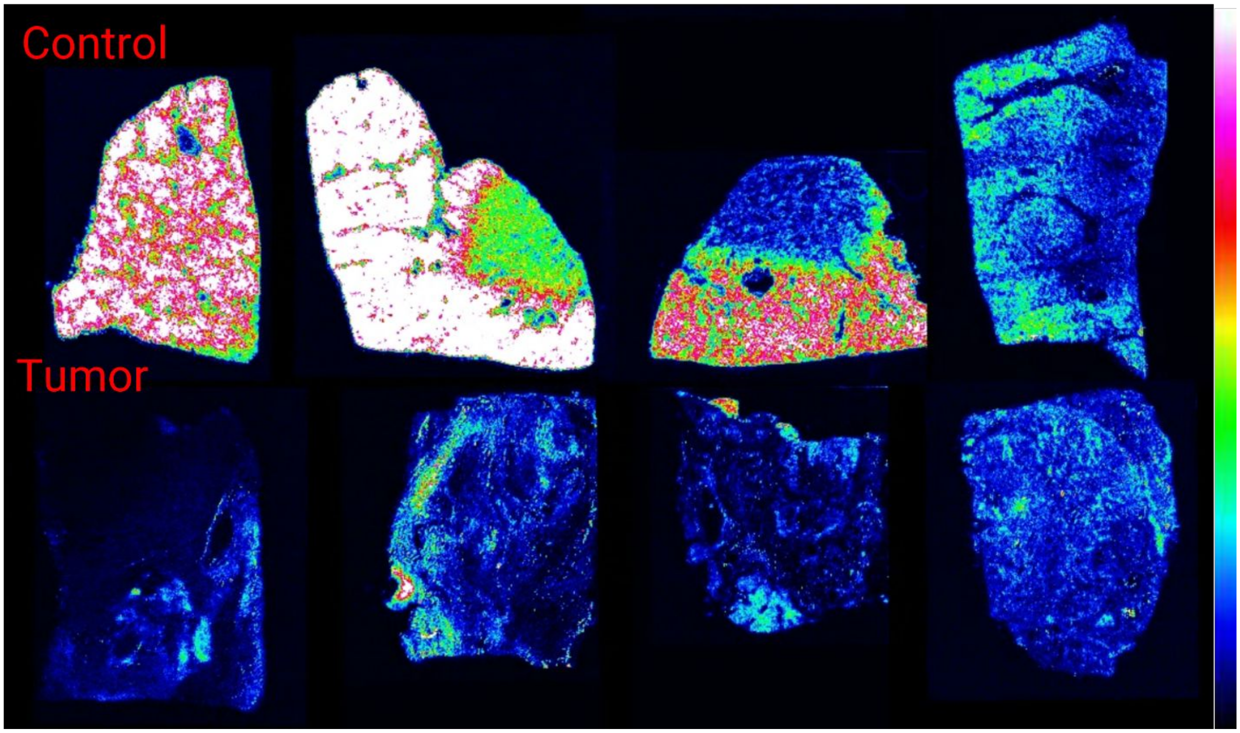

Figure S5. Rocuronium Ion Image in All Kidney Samples. The top four images display the control kidney samples, while the tumor pairs are shown at the bottom. Each control-tumor pair is positioned top to bottom. These images indicate that rocuronium accumulates in control kidney samples but not in the tumor pairs, suggesting that rocuronium cannot penetrate fatty tumor tissue.

Table S1. Differential Renal Cell Carcinoma Analytes. The 70 putatively annotated differential metabolites. Many PC, PE and TG were LC-MS/MS confirmed.

FC (Tumor/ Control)	P-value	*m/z*	ID	Adduct	Mass error (ppm)	CCS (Lin & Costello; Å²)	CCS (Instrument Default; Å²)	Drift Time (bins)
0.27	0.077	504.3052	LPE 18:0	[M+Na]+	-1.8	230.63	228.05	85.14
0.34	0.042	520.3374	LPC 18:2	[M+H]+	-4.6	236.54	228.48	85.63
0.27	0.035	522.3539	LPC 18:1	[M+H]+	-2.9	236.44	233.24	89.36
0.24	0.010	534.2947	LPC 16:0	[M+K]+	-1.7		234.69	90.6
0.28	0.086	544.3357	LPC 18:1	[M+Na]+	-3.1	238.29	234.16	90.28
0.30	0.014	560.3103	LPC 18:1	[M+K]+	-1.8		237.39	92.94
4.46	0.030	639.4938	DG 36:4	[M+Na]+	-3.3		264.44	114.71
7.92	0.037	645.5578	CE 16:1	[M+Na]+	-0.5		287.80	133.06
4.89	0.037	665.5088	DG 38:5	[M+Na]+	-4.1		269.31	118.71
6.33	0.007	667.5248	DG 38:4	[M+Na]+	-3.6	273.10	272.58	121.29
5.46	0.057	683.499	DG 38:4	[M+K]+	-3.1		272.24	121.13
0.38	0.096	716.5197	PE 34:2	[M+H]+	-3.9	276.99	276.86	124.97
0.45	0.083	718.5354	PE 34:1	[M+H]+	-3.8	279.04	280.07	127.5
0.31	0.015	738.5028	PE 34:2	[M+Na]+	-2.2	280.77	282.41	129.46
0.29	0.040	740.5193	PE 34:1	[M+Na]+	-1.1	283.32	282.17	129.28
0.34	0.001	746.5078	PE O-36:5	[M+Na]+	-2.3	284.13	285.91	132.26
0.33	0.009	758.5656	CL 76:3	[M+2H]2+	2.4		467.88	93.14
0.48	0.028	758.5681	PC 34:2	[M+H]+	-1.7	287.73	290.88	136.23
0.57	0.007	766.535	PE 36:2	[M+Na]+	-0.9	286.56	288.12	134.11
0.20	0.051	778.4769	PE 36:4	[M+K]+	-1.9	285.53	287.59	133.76
0.61	0.046	778.5345	PC 34:3	[M+Na]+	-1.5		290.43	135.99
0.41	0.001	780.5504	PC 34:2	[M+Na]+	-1.3	290.05	293.73	138.6

0.38	0.011	781.5562	CL 80:8	[M+2H]2+	0.3		467.09	92.89
0.39	0.086	782.5645	CL 80:7	[M+2H]2+	0.9		469.07	93.67
0.50	0.058	782.5652	PC 34:1	[M+Na]+	-2.3	292.05	296.06	140.44
0.56	0.064	784.5806	PC 34:0	[M+Na]+	-2.7	292.36	296.39	140.71
0.56	0.009	788.5163	PE 38:5	[M+Na]+	-4.8		290.54	136.13
0.46	0.001	790.5366	PE 38:4	[M+Na]+	1.1	290.98	295.10	139.73
0.42	0.011	792.5497	CL 82:11	[M+2H]2+	1.9		466.89	92.83
0.40	0.023	793.5568	CL 82:10	[M+2H]2+	1.0		468.15	93.33
0.45	0.077	794.5645	CL 82:9	[M+2H]2+	0.9		469.66	93.94
0.41	0.020	796.5253	PC 34:2	[M+K]+	0.0	290.67	294.55	139.33
0.46	0.020	798.5402	PC 34:1	[M+K]+	-1.0	293.27	298.52	142.46
0.45	0.022	804.55	PC 36:4	[M+Na]+	-1.7	293.56	298.20	142.24
0.40	0.016	805.5562	CL 84:12	[M+2H]2+	0.2		470.30	94.22
0.48	0.016	806.5644	PC 36:3	[M+Na]+	-3.2	294.01	298.69	142.64
0.43	0.041	806.5646	CL 84:11	[M+2H]2+	1.0		471.68	94.77
0.43	0.019	820.5251	PC 36:4	[M+K]+	-0.2		298.37	142.46
0.47	0.016	822.5391	PC 36:3	[M+K]+	-2.3		299.28	143.19
0.62	0.077	824.5564	PC 36:2	[M+K]+	-0.2	296.66	302.65	145.85
0.56	0.019	828.5263	PC O-38:7	[M+K]+	-4.9		298.77	142.82
0.47	0.007	844.5215	PC 38:6	[M+K]+	-4.5		300.76	144.46
0.59	0.035	846.5387	PC 38:5	[M+K]+	-2.7		302.83	146.1
15.70	0.061	855.7399	TG 50:1	[M+Na]+	-1.5		333.07	169.96
13.14	0.057	877.7236	TG 52:4	[M+Na]+	-2.3		332.24	169.42
11.84	0.034	879.7391	TG 52:3	[M+Na]+	-2.4		334.81	171.46

11.69	0.016	881.7543	TG 52:2	[M+Na]+	-2.9		337.38	173.49
0.22	0.022	885.6714	Coenzyme Q10	[M+Na]+	-1.9		316.50	157.06
15.37	0.037	885.7851	TG 52:0	[M+Na]+	-3.5		339.18	174.93
0.12	0.009	901.6487	Coenzyme Q10	[M+K]+	1.8		317.87	158.21
11.48	0.034	901.7225	TG 54:6	[M+Na]+	-3.4		336.54	172.93
18.85	0.013	903.7388	TG 54:5	[M+Na]+	-2.7		338.38	174.39
15.83	0.018	905.7544	TG 54:4	[M+Na]+	-2.8		340.21	175.84
14.29	0.014	907.7699	TG 54:3	[M+Na]+	-2.9		342.38	177.56
14.54	0.011	909.7842	TG 54:2	[M+Na]+	-4.4		344.75	179.44
10.00	0.034	925.7229	TG 56:8	[M+Na]+	-2.9		341.19	176.71
11.52	0.042	925.7646	TG 54:2	[M+K]+	2.7		345.82	180.36
10.09	0.022	927.7374	TG 56:7	[M+Na]+	-4.1		342.55	177.79
14.14	0.010	929.7523	TG 56:6	[M+Na]+	-4.9		345.27	179.94
29.59	0.002	931.7689	TG 56:5	[M+Na]+	-3.9		347.64	181.82
27.26	0.004	933.7836	TG 56:4	[M+Na]+	-4.9		348.79	182.74
19.45	0.017	935.8015	TG 56:3	[M+Na]+	-2.5		350.85	184.37
15.13	0.038	937.8165	TG 56:2	[M+Na]+	-3.2		353.90	186.79
22.60	0.056	947.7473	TG 56:5	[M+K]+	0.9		348.06	182.23
22.80	0.029	949.7618	TG 56:4	[M+K]+	-0.3		350.12	183.86
17.25	0.013	957.7842	TG 58:6	[M+Na]+	-4.2		353.86	186.85
22.58	0.001	959.8004	TG 58:5	[M+Na]+	-3.5		355.16	187.88
18.20	0.035	961.8151	TG 58:4	[M+Na]+	-4.6		356.69	189.1
11.61	0.023	971.7429	TG 58:7	[M+K]+	-3.6		351.59	185.12
16.47	0.011	973.7605	TG 58:6	[M+K]+	-1.6		354.33	187.29
